# A comprehensive approach to evaluate genetic abnormalities in multiple myeloma using optical genome mapping

**DOI:** 10.1038/s41408-024-01059-x

**Published:** 2024-05-03

**Authors:** Ying S. Zou, Melanie Klausner, Jen Ghabrial, Victoria Stinnett, Patty Long, Laura Morsberger, Jaclyn B. Murry, Katie Beierl, Christopher D. Gocke, Rena R. Xian, Kevin H. Toomer, Jing Christine Ye, Robert Z. Orlowski, Carol Ann Huff, Syed Abbas Ali, Philip H. Imus, Christian B. Gocke, Guilin Tang

**Affiliations:** 1 Department of Pathology, Johns Hopkins University School of Medicine, Baltimore, MD USA; 2 Department of Lymphoma & Myeloma, The University of Texas MD Anderson Cancer Center, 1515 Holcombe Blvd, Houston, TX 77030 USA; 3 Department of Experimental Therapeutics, The University of Texas MD Anderson Cancer Center, 1515 Holcombe Blvd, Houston, TX 77030 USA; 4 Department of Oncology, The Sydney Kimmel Comprehensive Cancer Center, Johns Hopkins University School of Medicine, Baltimore, MD USA; 5 Department of Hematopathology, The University of Texas MD Anderson Cancer Center, 6565 MD Anderson Blvd, Houston, TX 77030 USA

**Keywords:** Cytogenetics, Cancer genetics

Dear Editor,

Multiple myeloma (MM) is a plasma cell neoplasm (PCN) [1]. Sensitive and accurate identification of genetic abnormalities is critical for patient risk stratification and therapeutic decision-making as well as understanding pathogenesis. Recurring genetic abnormalities include structural variants (SVs), such as rearrangements involving *IGH* and *MYC* genes; copy number variants (CNVs), such as 1q+, del(17p), del(13q); hyperdiploidy/hypodiploidy; and various gene mutations, such as mutations in the *RAS* pathway, *BRAF, FAM46C, DIS3*, and *TP53* [2]. Trisomies/hyperdiploidy and *IGH* rearrangements/fusions are considered primary abnormalities present at disease initiation while 1q+, del(17p)/*TP53* mutations and *MYC* rearrangements/fusions are considered secondary abnormalities that develop during disease progression [3]. High-risk genetic abnormalities including del(17p), *t*(4;14)/*IGH::FGFR3*/*NSD2*, and *t*(14;16)/*IGH::MAF* have been used along with serum markers for MM risk stratification in the Revised International Stage System [4]. Other genetic abnormalities including *t*(14;20)/*IGH::MAFB*, and *MYC* rearrangements/fusions are considered potential high-risk biomarkers in MM [2]. Recently whole-genome sequencing (WGS) studies have revealed novel candidate driver genes and catastrophic complex rearrangements (chromoanagenesis) associated with poor clinical outcomes [5]. Furthermore, the presence of some genetic abnormalities can clearly impact response to specific therapies, as exemplified by *t*(11;14)/*IGH::CCND1* and response to BCL2 inhibition [2, 3, 6]. Therefore, detection of genome-wide genetic abnormalities is essential.

Detection of genetic abnormalities has routinely relied on karyotyping, fluorescence in-situ hybridization (FISH), and next-generation sequencing (NGS). Karyotyping offers a low-resolution whole-genome analysis and requires the presence of actively dividing cancer cells [7]. FISH is more sensitive than karyotyping within targeted areas covered by probes but still leaves most of the genome unexamined [7]. Furthermore, complex gene rearrangements can make interpretation and analysis of FISH difficult. For example, FISH failed to detect ~70% of all *MYC* SV subtypes reported by NGS [8] and variant *IGH* rearrangements may mask *IGH* rearrangements or cause equivocal results [9]. Chromosomal microarray (CMA) testing has been reported to provide a whole-genome analysis and improve diagnostic yield for CNVs compared to FISH/karyotyping [10]. However, CMA is not capable of detecting balanced SVs (e.g., *IGH* or *MYC* rearrangements/fusions), which limits its clinical utility. Currently, RNA-sequencing and WGS show promising genetic data in MM [5]. These approaches require complex bioinformatics pipelines for the detection of SVs/CNVs, an abundance of CD138+ plasma cells, and large capital equipment requirements, which may be challenging to establish in a clinical laboratory.

Optical genome mapping (OGM) is an emerging technology that uses fluorescently labeled ultra-high molecular weight genomic DNA to restructure genome-wide SV and CNV maps. It has a simple workflow and straightforward bioinformatics analysis pipelines. It has been shown to detect genome-wide SVs/CNVs in hematologic malignancies [11–13]. To date, two pilot studies of OGM on CD138+ plasma cells have been reported: one study compared OGM data between 4 extramedullary and 7 intramedullary MM [14] and the other compared OGM and FISH results in 20 MM cases with plasma cell percentages of ≥10% [15]. Although both studies showed promising OGM results in MM for detection of genome-wide SVs and CNVs, there is no multi-center study reporting the clinical utility of combined OGM and NGS for pathogenesis/prognostication of MM and other PCN (with plasma cell percentages of <10%) in clinical practice.

This study includes concurrent karyotyping, FISH, OGM, and NGS analyses on 45 PCN patients from The Johns Hopkins Hospital and The University of Texas MD Anderson Cancer Center from January 2022 to November 2023 (Table 1, see Materials and Methods in the supplementary file for details). It includes 35 MM, 6 smoldering myeloma, 2 monoclonal gammopathy of undetermined/renal significance, and 2 amyloid light chain (AL) amyloidosis (Supplementary Table S1). One patient has simultaneous diagnosis of both MM and myelodysplastic syndrome (MDS) (case #30). OGM was performed on CD138+ plasma cells for cases #1–30 with median 25% plasma cells by morphology, and on fresh biopsy/aspirate for cases #31–45 without CD138-enrichment having >50% plasma cells. All FISH and NGS were performed on CD138+ uncultured specimens. The specimen was considered abnormal based on laboratory-established cutoffs/criteria. Concordance of karyotyping/FISH and OGM results was determined by a systematic review of the loci of interest for each sample. This study was approved by the Institutional Review Board and performed in accordance with the Declaration of Helsinki.

**Table 1:** Genetic abnormalities by karyotyping, FISH, OGM, and NGS.

ID^a^	Sex	Age	PC % core/diff (flow)^b^	Karyotype	FISH abnormality	OGM for FISH loci	OGM additional CNVs & SVs (Tier 1/2)	Pathogenic gene mutation by NGS
1	F	55	25% (5.3%)	Normal	*IGH::CCND1*	Yes	−X, −13, and 11q+	No pathogenic mutations
2	M	77	13% (2.5%)	Normal	1q22+	Yes	Hyperdiploidy, -X, −20, 2p+^d^	*IKZF3* p.H474P
3	M	65	13% (0.5%)	Normal (−Y)	*IGH::MAF*	Yes	−Y	No pathogenic mutations
4	F	68	55% (7.3%)	N/A	1p36.3−, 1q22+	Yes	Hypodiploidy, 9q+	No pathogenic mutations
5	F	48	75% (5.6%)	Abnormal	1q22+, *IGH* sep., *MAF−*, *MAFB−*	Yes (*IGH::MAFA*^c^)	13q−^d^	No pathogenic mutations
6	F	67	90% (46.3%)	Abnormal	1q22+, *IGH::CCND1*	Yes	−13, +15, chromoanagenesis, 1p−^d^	*KRAS* p.G12A, *NFKBIZ* p.E122fs
7	M	76	25% (17.8%)	Normal	*IGH::CCND1*, *TP53−*	Yes	−Y, −13, 11q+, 16q−, *CD38*−	*IDH1* p.R132L, *KRAS* p.Q61H, *DIS3* p.G948^a^, *TP53* p.K373fs
8	M	76	5% (1.5%)	Normal	1q22+, *IGH::MAF*, *CCND1*+	Yes	Hyperdiploidy, −13, 20p−, −22	N/A
9	M	72	5% (0.6%)	Normal	Normal	Yes	Hyperdiploidy, −13	N/A
10	F	51	50% (11.5%)	N/A	Equivocal *IGH* sep., *MAF−*	Yes (*IGH::MYC*^c^)	Hyperdiplody, −X, −13, 16q−	*KRAS* p.G60D, *TENT5C* p.K82fs & p.H96fs, *PTPN11* p.N308D
11	F	69	55% (0.8%)	Normal	*TP53−*	Yes	*IGH::MAFB*, hypodiploidy, 1p−, 5p−, 5q−, 22q−	N/A
12	F	66	13% (0.3%)	Normal	*IGH::MAF*	Yes	−X, +4, +9, +16, +21, *TNFRSF17*+	No pathogenic mutations
13	M	74	65% (31.1%)	N/A	1q22+, *TP53*−	Yes	Hyperdiploidy, −Y, 10p−, 17q+, *TNFRSF17*+, chromoanagenesis	No pathogenic mutations
14	M	63	45% (4.8%)	Normal	*IGH* rearrangement	Yes (*IGH::CCND3*^*c*^)	*MYC::IGL*, Xq+, 1p−, 14q−, 15q+	No pathogenic mutations
15	M	75	15% (1.3%)	Normal (−Y)	*MYC*+, *TP53−*	Yes	Hyperdiploidy, −Y, 6q−, 8p−, 8q+, 17q+	*KRAS* p.Q61H
16	F	63	50% (20.8%)	Normal	*MYC* rearrangement	Yes (inverted *MYC*)	Hyperdiploidy, −X, −18, chromoanagenesis, *CD38*+^d^	*NRAS* p.Q61R
17	F	75	8% (0.7%)	N/A	1q22+	Yes	−X, −13, +15, 1p−	*IDH2* p.R140Q
18	M	73	<10% (0.3%)	Normal	*IGH::CCND1*	Yes	None	No pathogenic mutations
19	M	65	85% (21%)	Normal	*IGH::CCND1*	Yes	Hyperdiploidy, −13, 10q+, 12p−, 16q−	*KRAS* p.Q61H & p.G12C, *PIK3R2* p.P121S, *RB1* p.E440K & p.N505T, *CCND1* p.I132M
20	M	67	45% (N/A)	N/A	1q22+, *IGH::CCND1*	Yes	Hyperdiploidy, −Y, −13, 3q+, 11q+, *TNFRSF17*+, *MYC* sep.	*KRAS* p.Q61H, *IKZF3* p.Y202D
21	M	74	35% (N/A)	N/A	1q22+, *IGH*−	Yes	−Y, −13, −14, bi-allelic *RB1*−^d^	No pathogenic mutations
22	F	85	80% (32.8%)	N/A	1q22+, *IGH::MAF*	Yes	−13, −22, 1p−^d^	N/A
23	M	63	25% (4.0%)	Normal (−Y)	*IGH−*	Yes	Hyperdiploidy, Yq+, 14q−	N/A
24	M	87	20% (11.1%)	Normal	Normal	Yes	Hyperdiploidy, chromoanagenesis 1, 11, 1p−^d^	No pathogenic mutations
25	F	39	8% (1.6%)	Normal	*IGH−*	Yes	Hypodiploidy	No pathogenic mutations
26	M	89	65% (19.1%)	Normal	1q22+, *IGH::CCND1*	Yes	−Y, 6q−, chromoanagenesis	No pathogenic mutations
27	M	71	13% (0.6%)	Normal	Normal	Yes	Hyperdiploidy, 1p−, 6p+	*DIS3* p.D487H
28	M	57	20% (4.2%)	Normal	Normal	Yes	Hyperdiploidy, 6q−, 16q−, *MYC::IGL*, chromoanagenesis	*NRAS* p.Q61L
29	M	57	60% (14%)	N/A	Normal	Yes	Hyperdiploidy, Xq+	N/A
30	M	86	20% (0.9%)	Abnormal	*IGH::CCNDI*	Yes	−7, +8, −5q, dic (12; 17) (p13.32; p13.3)	*TP53* p.R213G
31	M	41	55% (4.6%)	Abnormal	1q22+, *MYC*−	Yes (except 1q+)	*IGH::FGFR3/NSD2*	No pathogenic mutations
32	M	66	62% (14%)	Normal	*CCND*+, +9	Yes	Hyperdiploidy, −Y, 6q−	N/A
33	M	70	67% (23%)	Abnormal	*CKS1B*+*, RB1*−	Yes	+3, +7, +22, *MYC::PECAM1*	N/A
34	F	66	93% (21%)	Abnormal	*CKS1B*+, *FGFR3*+*, IGH*+, *MYC*+, +9, *CCND1*+, *MAF::IGH*, *TP53−*	Yes	−(X, 7, 11, 13, 16, 22), +(3, 6, 8, 9, 18, 20), chromoanagenesis 1, 2, 12	*DNMT3A* c.2711C > T
35	M	74	70% (34%)	Abnormal	*FGFR3*+, *MYC* sep., +9, *CCND1*+, *MAF−*	Yes (*IGL::MYC*^c^)	Hyperdiploidy, 18q+, chromoanagenesis 13, 16, 18	N/A
36	F	57	75% (32%)	Normal	*IGH::CCND1, CKS1B*+, −13	Yes	+15, −X, *MYC::TENT5C*	*BCL7A* c.92+1G > C, *NRAS* p.Q61H
37	F	74	57% (5%)	Normal	*IGH::MAF, TP53−*, −13	Yes (except *TP53*−)	−X, *TMEM200C* sep., *ARIH2* sep.	*ATM* p.Q2637^a^ & p.K2440E, *BRAF* p.D594N, TP53 p.R337C, KLHL6 p.E17^a^, *KRAS* p.G13D, *PIK3CA* p.H665Y, PTPN1 c.494C > T, *PLCG2* p.S1029C
38	M	79	94% (40%)	Abnormal	*IGH::CCND1*, 5′*MYC* amp	Yes	9p−	N/A
39	M	63	74% (28%)	Abnormal	*IGH::CCND1*, *CDKN2C−*, *CKS1B*+, *MAF−*, *RB1*−	Yes	None	N/A
40	M	78	75% (81%)	Abnormal	*CKS1B*+, *MYC*+, *CDKN2A−*, +9, *CCND*+, −13, *MAF*−	Yes	Hyperdiploidy, *IGH::HDAC9*, *STK11* sep., 12p−^d^	*DH2* p.V305M, *KLF2* p.P125H, *NRAS* p.A59D
41	M	70	52% (26%)	Normal	*IGH::CCND1*	Yes	None	*ASXL1* p.G646fs, *CCND1* p.K46Q, *CDKN2B* c.158_ 159dupTC, *IGLL5* p.S47G, p.A32D, c.1A > G (<5) & p.S59N, *PLCG2* p.K381M, *TP53* p.A276G
42	F	68	71% (40%)	Abnormal	*FGFR3−*, *IGH−*, *CCND*+, +9	Yes	Hyperdiploidy, 1p−, *PTGFR::CD53*, *AL 606923.2* sep., *KIAA1109::FOXN3*, *AP3B1::EDIL3*, *ELP4::AC131571.1*, *DIAPH2* sep., *MACC1::ETV4*^d^	*IRAK1* p.N345S, *NF1* p.A706V, *RBMX* p.A66V, p.R298^a^ & p.R6C, *TENT5C* p.L142P
43	F	76	78% (89%)	Abnormal	*CKS1B*+, *IGH::FGFR3/ NSD2, MYC* sep., *CDKN2A−*, −13, *IGH*+	Yes (*MYC::NBEA*^c^)	None	N/A
44	M	49	79% (35%)	Abnormal	−1, *TP53−*, *MYC−*, −13, *IGH*−, *MAF−*	Yes	Hypodiploidy, 6q−	N/A
45	M	56	58% (53%)	Abnormal	*CKS1B*+, *CCND1::IGH*, −13	Yes	1p−^d^, numerous intra- and inter-chromosomal rearrangement	*DNMT3A* p.R729W & p.L566^a^, *GNA13* p.C18S, *IGLL5* p.A54V, *SOX11* p.D233E

NGS performed in CLIA/CAP-certified molecular diagnostics labs with coverage (>250×) and mutant allele frequency (>5%).

*sep.* rearrangement, + gain, − deletion/loss, *N*/*A* not available.

^a^OGM performed on CD138+ cells for sample IDs 1–30 and on fresh biopsy/aspirate without CD138+ for samples IDs 31–45.

^b^For sample IDs 1–31, % PC reported for core biopsy from surgical pathology report. For sample IDs 32–45, % PC reported based on differential results.

^c^OGM revealed translocation partners.

^d^≥5 additional copy number variants (gains >10 Mb & losses >5 Mb) besides monosomy or trisomy.

In this cohort, 14 cases (38%) had abnormal karyotypes and a total of 233 genomic loci were tested by FISH with an average of 5 FISH loci per specimen (Supplementary Table S1). Ninety-eight percent of these loci (229 out of 233) displayed concordance between FISH and OGM (Table 1). Four discordant loci demonstrated the following: equivocal *IGH* rearrangement by *IGH* break-apart FISH had *IGH::MYC* fusion by OGM (case #10); *MYC* rearrangement by FISH had complex nested inversions within 8q by OGM (case #16); and two low-level CNVs by FISH were undetected by OGM due to low levels of plasma cells (cases #31 and #37 – both unselected cases with <5% plasma cells by flow) (Supplementary Fig. S1). Compared with FISH loci, OGM achieved 100% sensitivity, specificity, and accuracy in CD138+ cases and 96.6% sensitivity, 100% specificity, and 98.3% accuracy in unselected cases. Furthermore, OGM identified potential translocation partners in 5 cases, which supported *IGH* and/or *MYC* rearrangements by FISH (*IGH::MAFA* in case #5, *IGH::MYC* in case #10, *IGH::CCND3* in case #14, *IGL::MYC* in case #35, *MYC::NBEA* in case #43). OGM identified two *t*(4; 14) samples with *IGH::FGFR3*/*NSD2* fusions (cases #31 and #43).

Compared to limited FISH loci, OGM provided a genome-wide profile of SVs and CNVs. OGM revealed 18 hyperdiploidy, 4 hypodiploidy, and 9 *IGH* or *MYC* rearrangements that were not tested by FISH, as well as 8 cases (18%) with chromoanagenesis (complex genomic rearrangements and copy number alterations) (Table 1). OGM changed the prognostication beyond standard cytogenetics/FISH testing in eight cases (18% of cases in this study). Most samples displayed additional SVs/CNVs (Tier 3) by OGM: on average, an additional 7 intra- and inter-chromosomal translocation events (ranging from 0 to 90 translocations with a median of 4), an additional 0–5 CNV gain events (>1 Mb), and 0–14 CNV loss events (>1 Mb) per sample (Supplementary Table S2).

OGM’s comprehensive genome-wide SVs/CNVs profile led to classifying genetic subtypes in MM (Fig. 1). All cases had known MM abnormalities including 24 with *IGH* gene fusions, 15 with hyperdiploidy, and 6 had other abnormalities. These cases can be further classified according to additional MM abnormalities. Chromoanagenesis was frequent in cases with hyperdiploidy (*n* = 4, 27% of all hyperdiploidy cases) and in cases with *IGH::CCND1* (*n* = 3, 23% of all *IGH::CCND1* fusion cases). *MYC::IGL* fusions and *NRAS* mutations were found in half of the cases with chromoanagenesis and hyperdiploidy (cases #28, #35, and cases #16, #28, respectively). Gain of 1q was found in all cases with chromoanagenesis and *IGH* rearrangements/fusions, which might be a novel molecular subtype. Concurrent myeloma NGS results detected pathogenic mutations in 29 cancer genes, with recurrent mutations in *KRAS* (27%)*, IGLL5, NRAS, TENT5C, TP53* (10%) and *CCND1, CDKN2B, DIS3, DNMT3A, IDH1/2, IKZF3, and PLCG2* (7%).

**Fig. 1:**
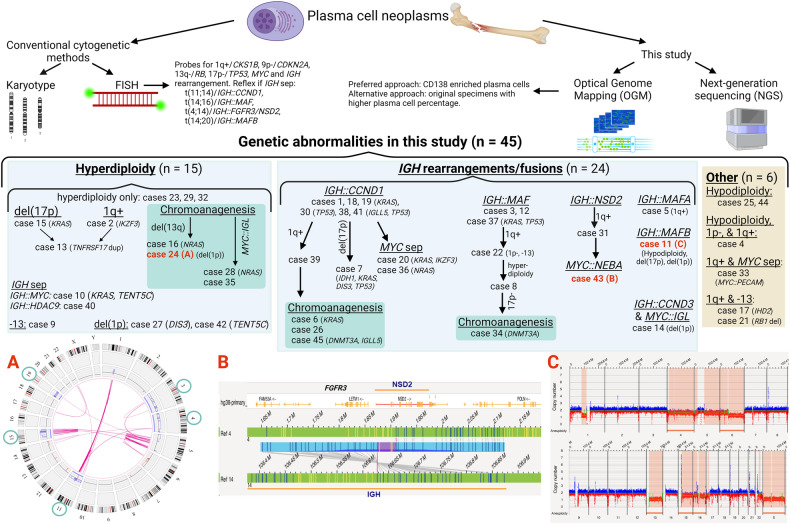
Potential genetic testing workflow to classify PCN genetic subtypes in a clinical setting. OGM and NGS on CD138+ plasma cells are preferred, although alternative approaches include OGM and NGS on original specimens with a higher plasma cell burden. **A** Circos plot of case #24 showing chromoanagenesis (red lines in the center) and hyperdiploidy (gain of chromosomes 3, 4, 11, 15, and 19, green circles). **B** Breakpoints of case #43 showing an *IGH::FGFR3/NDS2* fusion. **C** Whole-genome view of case #11 shows hypodiploidy with losses of chromosomes 4, 6, 13, 15, 16, X, del(17p), and partial del(1p). Common pathogenic mutations in this cohort are listed for each case.

Given that only approximately one-third of cases had abnormal karyotypes and that FISH included assessment for a limited number of loci, OGM allowed for a more comprehensive definition of the plasma cell genome, not only for well-established FISH targets/regions, but also for hyperdiploidy/hypodiploidy, chromoanagenesis, atypical *IGH* and/or *MYC* translocation partners, and 366 novel SVs/CNVs that might be important in the formation and development of MM (Supplementary Tables S1–2). For case #30 with both MM and MDS, MM FISH on CD138+ plasma cells detected an *IGH::CCND1* fusion while MDS FISH panel and karyotype on the fresh bone marrow detected del(5q), −7, +8, and dic(12p; 17p). OGM was able to detect both the MM and MDS abnormalities.

Genome-wide SVs/CNVs by OGM may be therapeutically helpful. For example, gain of *BCMA/TNFRSF17* on 16p13.13 was found in patients #12 (+16) and #13 and #20 (*BCMA/TNFRSF17*+), detecting *BCMA* CNVs, such as gain/amplification or bi-allelic loss, may be associated with the effectiveness or resistance of BCMA-targeted monoclonal antibodies or CAR T-cell therapies. Patients #19 and #40 had a loss of *GPRC5D* on 12p13.1, who may not be ideal candidates for GPRC5D-targeted therapies.

In this study, genome-wide OGM analysis facilitated classification of genetic subtypes in PCN. We propose a potential clinical workflow for diagnostic testing for PCN (Fig. 1). We advise to perform OGM and NGS on CD138+ plasma cells. Genome-wide OGM results will not only provide comprehensive SVs/CNVs landscape for clarifying cytogenetic risks, but also may lead to the discovery of novel genetic biomarkers. In the absence of complex bioinformatics pipelines, OGM is an emerging method for genome-wide detection of SVs/CNVs in PCN.

In conclusion, this is the first and largest study (performed at two academic centers) reporting the value of combined OGM and NGS for PCN pathogenesis in clinical practice. A combination of OGM for genome-wide SVs/CNVs and NGS to interrogate for gene mutations may become an essential approach for evaluating genetic abnormalities in MM in the clinical setting. Future multi-center studies that incorporate larger numbers of MM cases, obtain comprehensive clinical data, and follow various treatment strategies will shed light on how genetic subtypes of MM are related to treatment response rates, survival, and overall prognosis.

### Supplementary information

Supplemental Table S1

Supplemental Table S2

Supplementary Methods and Supplementary Figure S1

## Data Availability

The datasets generated during and/or analyzed during the current study are available from the corresponding author on reasonable request.
