# Supplementary material for: A comprehensive approach to evaluate genetic abnormalities in multiple myeloma using optical genome mapping
Source: Blood Cancer J. 2024 May 3;14(1):78. doi: 10.1038/s41408-024-01059-x (PMC11068911; doi:10.1038/s41408-024-01059-x)
Supplement: Supplementary file 1 — Supplemental Table S1 [file 41408_2024_1059_MOESM1_ESM.docx]

* For sample IDs 1-30, OGM performed on CD138 enriched cells. For samples IDs 31-45, OGM performed on fresh specimens without enrichment. AL=immunoglobulin light-chain amyloidosis; MGRS=monoclonal gammopathy of renal significance; MGUS=monoclonal gammopathy of undetermined significance; NDMM=newly diagnosed multiple myeloma; RRMM=refractory/relapsed multiple myeloma.
