# Supplementary material for: A comprehensive approach to evaluate genetic abnormalities in multiple myeloma using optical genome mapping
Source: Blood Cancer J. 2024 May 3;14(1):78. doi: 10.1038/s41408-024-01059-x (PMC11068911; doi:10.1038/s41408-024-01059-x)
Supplement: Supplementary file 2 — Supplemental Table S2 [file 41408_2024_1059_MOESM2_ESM.docx]

* For sample IDs 1-30, OGM performed on CD138 enriched cells. For samples IDs 31-45, OGM performed on fresh specimens without enrichment.
