# Supplementary material for: A comprehensive approach to evaluate genetic abnormalities in multiple myeloma using optical genome mapping
Source: Blood Cancer J. 2024 May 3;14(1):78. doi: 10.1038/s41408-024-01059-x (PMC11068911; doi:10.1038/s41408-024-01059-x)
Supplement: Supplementary file 3 — Supplementary Methods and Supplementary Figure S1 [file 41408_2024_1059_MOESM3_ESM.docx]

**The supplemental file of Materials and Methods**

**Patients and Samples**

This study included 45 patients with plasma cell neoplasm, who were referred to The Johns Hopkins Hospital and The University of Texas MD Anderson Cancer Center from January 1, 2022, to November 30, 2023. These patients had routine diagnostic procedures, including morphologic evaluation, flow cytometry, fluorescence *in situ* hybridization (FISH), conventional chromosome analysis, and/or a targeted next-generation sequencing (NGS) assay. Disease classification by standard hematopathology practice and delineated by the World Health Organization was based on clinical, morphologic, immunophenotypic, cytogenetic, and molecular genetic features.

**Conventional Chromosome Analysis and Fluorescence *in Situ* Hybridization (FISH)**

Conventional G-banded chromosome studies were performed using standard techniques. A minimum of 20 metaphase cells were analyzed from unstimulated bone marrow aspirate. The abnormal karyotypes were described using the International System for Human Cytogenetic Nomenclature (2020).

FISH panel testing was performed on CD138+ plasma cells. 233 FISH was performed on interphase nuclei using disease-specific panels of probes, according to the manufacturer's protocol (Abbott Molecular Inc., Des Plaines, IL). The specimen was considered abnormal if the results exceeded the laboratory-established cutoff for each probe set.

**Optical genome mapping (OGM)**

OGM was performed on CD138+ plasma cells (cases 1 through 30) and on fresh biopsy/aspirates (cases 31 through 45). CD138 cell selection was performed on bone marrow samples via manual isolation utilizing CD138 microbeads and autoMACS magnetic column from Miltenyi Biotec (Gaithersburg, MD, USA) or via the automated RoboSep instrument from StemCell Technologies (Cambridge, MA, USA). G3.3 chips were utilized, and samples were processed on the Bionano Saphyr instrument (San Diego, CA, USA). OGM analysis was performed using the Rare Variant Analysis (RVA) and De Novo (DN) pipelines, utilizing the Bionano Access software v1.7.2. CNVs and SVs were manually determined by two genetic analysts independently.

**Targeted Next-generation sequencing (NGS) mutation assay**

NGS performed in CLIA/CAP-certified molecular diagnostics labs and was performed on CD138+ plasma cells. CD138 cell selection was performed via the automated RoboSep instrument from StemCell Technologies (Cambridge, MA, USA). For cases 1 through 30, DNA concentration was assessed using the Qubit fluorometer (Thermo Fisher Scientific, Waltham, MA). Library preparation was performed using Kapa Roche (Wilmington, MA) reagents, hybrid capture was performed using Integrated DNA Technologies probes (Coralville, IA), and products were sequenced using NovaSeq (paired-end technology; Illumina, San Diego, CA). The targeted NGS assay used 40,670 Integrated DNA Technologies probes and for a list of covered cancer genes in the targeted NGS assay, see https://pathology.jhu.edu/jhml-services/assets/test-directory/Myeloma-Panel_GeneList_v1.0.pdf. Analysis was performed using human reference sequence genome assembly hg19 (NCBI build GRCh37/hg19). An in-house variant caller software (MDL VC 10) was used to generate gene variants/mutations from the targeted NGS data. For cases 31 through 45, NGS gene panel includes *ARID1A, ASXL1, ATM, B2M, BAZ2A, BCL10, BCL2, BCL6, BCL7A, BCOR, BIRC3, BLNK, BRAF, BRCC3, BTG1, BTG2, BTK, CARD11, CCND1, CCND3, CCR4, CCR7, CD274, CD28, CD58, CD79A, CD79B, CDKN2A, CDKN2B, CHD2, CHEK2, CIITA, CNOT3, CREBBP, CXCR4, DDX3X, DIS3, DNMT3A, DUSP2, EGR1, EGR2, ELF4, EP300, EWSR1, EZH2, FAM50A, FAS, FAT1, FBXW7, FGFR3, FOXO1, FYN, GNA13, GNAS, GPR183, H1-2, H1-4, H3C2, HRAS, HUWE1, HVNC1, ID3, IDH1, IDH2, IFNGR1, IGLL5, IKZF3, IL2RG, IRAK1, IRF4, IRF8, ITPKB, JAK1, JAK2, JAK3, KIT, KLF2, KLHL6, KMT2D, KRAS, LTB, LYN, MAP2K1, MAP3K14, MAPK1, MAX, MED12, MEF2B, MFHAS1, MYC, MYD88, NF1, NFKB2, NFKBIA, NFKBIE, NOTCH1, NOTCH2, NPM1, NRAS, NSD2, NXF1, P2RY8, PAX5, PIK3CA, PIK3R1, PIM1, PLCG1, PLCG2, PLEKHG5, POLE, POT1, PRDM1, PTEN, PTPN1, PTPN11, PTPRD, RASSF1, RB1, RBMX, RFTN1, RHOA, RIPK1, RPS15, RRAGC, RRAS, S1PR1, S1PR2, SAMHD1, SETD2, SF3B1, SGK1, SMARCA4, SMO, SOCS1, SOX11, SP140, SPEN, SRSF2, STAT3, STAT5B, STAT6, STK11, SYK, TBL1XR1, TCF3, TENT5C, TET2, TMEM30A, TNFAIP3, TNFRSF14, TP53, TRAF2, TRAF3, TRAF6, U2AF1, UBR5, VAV1, XPO1, ZFAT, ZMYM3,* and *ZRSR2*. NGS had coverage (>250x) and mutant allele frequency (>5%).

**Statistical calculators**

Comparison of sensitivity, specificity, and the accuracy of the OGM and FISH was performed using MEDCALC statistical software (https://www.medcalc.org/ calc/diagnostic_test.php, last accessed December 14, 2023).

**Supplemental Figure S1:**


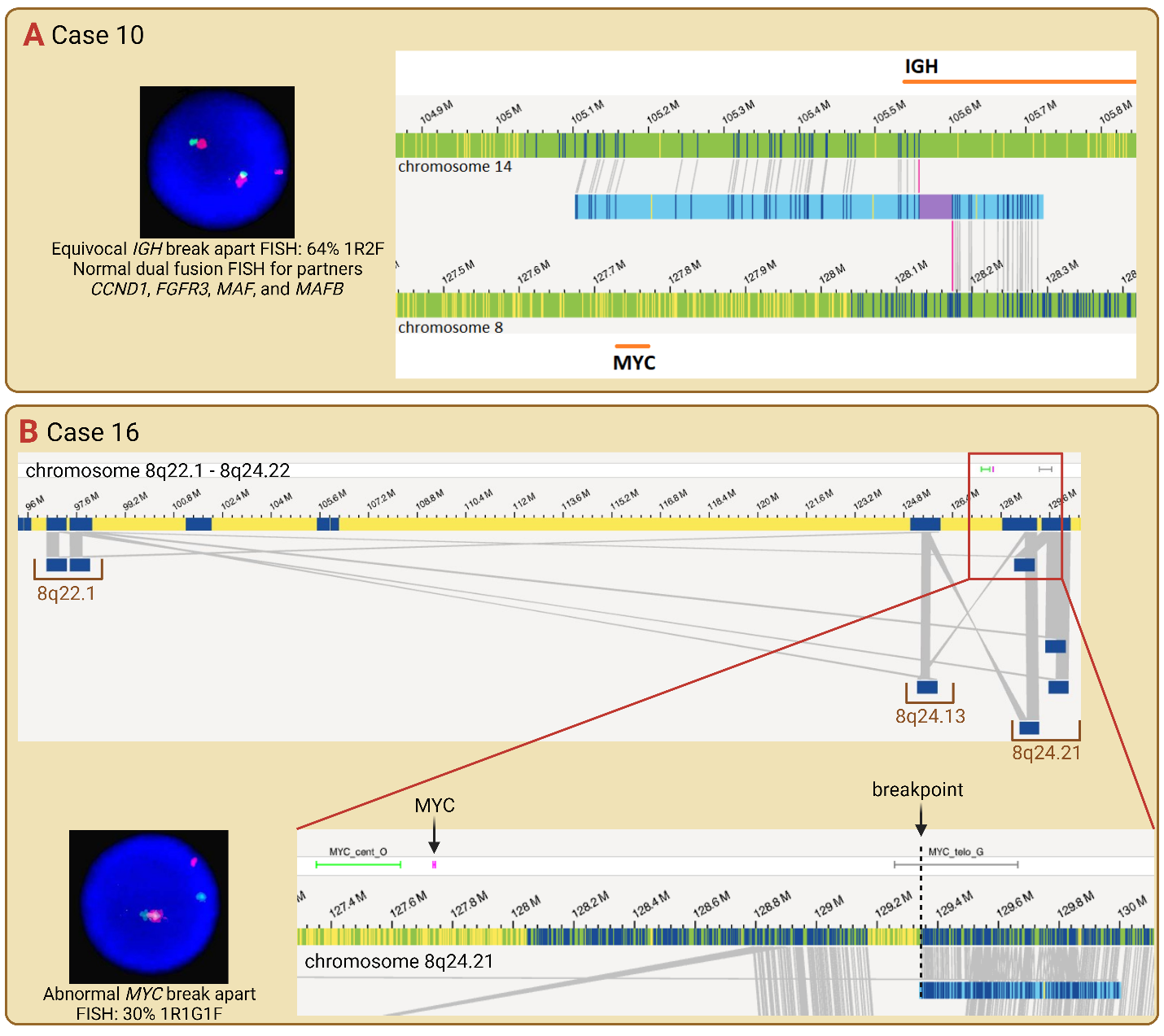


Supplemental Figure S1: Discordant cases of OGM and FISH. (A) Case #10: *IGH* break apart FISH reported as equivocal due to an atypical signal pattern (1R2F), possibly due to duplication, deletion, or expansion of a normal *IGH* variable region. Reflex to dual fusion FISH for *CCND1*, *FGFR3*, *MAF*, and *MAFB* partners were all normal. OGM detected a t(8;14)(q24.21;q32.33), a *IGH::MYC* gene fusion. (B) Case #16: While *MYC* break apart FISH displayed the typical abnormal pattern consistent with disruption of the *MYC* gene (1R1G1F), OGM identified a series of complex, nested inversions involving multiple locations on 8q, disrupting the green/telomeric portion of the *MYC* break apart FISH probe (“MYC_telo_G”, dotted line displays the breakpoint within that probe), despite *MYC* itself remaining apparently intact.
